# How Feeling Betrayed Affects Cooperation

**DOI:** 10.1371/journal.pone.0122205

**Published:** 2015-04-29

**Authors:** Pouria Ramazi, Jop Hessel, Ming Cao

**Affiliations:** ENgineering and TEchnology institute Groningen (ENTEG), Faculty of Mathematics and Natural Sciences, University of Groningen, Groningen, The Netherlands; Tianjin University of Technology, CHINA

## Abstract

For a population of interacting self-interested agents, we study how the average cooperation level is affected by some individuals' feelings of being betrayed and guilt. We quantify these feelings as adjusted payoffs in asymmetric games, where for different emotions, the payoff matrix takes the structure of that of either a prisoner's dilemma or a snowdrift game. Then we analyze the evolution of cooperation in a well-mixed population of agents, each of whom is associated with such a payoff matrix. At each time-step, an agent is randomly chosen from the population to update her strategy based on the myopic best-response update rule. According to the simulations, decreasing the feeling of being betrayed in a portion of agents does not necessarily increase the level of cooperation in the population. However, this resistance of the population against low-betrayal-level agents is effective only up to some extend that is explicitly determined by the payoff matrices and the number of agents associated with these matrices. Two other models are also considered where the betrayal factor of an agent fluctuates as a function of the number of cooperators and defectors that she encounters. Unstable behaviors are observed for the level of cooperation in these cases; however, we show that one can tune the parameters in the function to make the whole population become cooperative or defective.

## Introduction

For years, scientists have tried to close the gap between what theoretical game theory predicts and the actual outcome in game theoretic experiments with humans. Classical models in game theory, such as the model of Axelrod [1], predict that defection will be the dominant strategy where the *prisoner’s dilemma (PD)* game is played in a population of rational players. However, cooperative behaviour often does appear in real-life situations, as many social experiments with the prisoner’s dilemma and other public goods games have shown [2] [3] [4] [5] [6]. Previous research has proposed different mechanisms for the promotion of cooperation, e.g., inferring reputation [7], incorporating costly punishment [8], iterating the games [9] [10] [11], using multiplayer games [12], incorporating an interaction network [13] [14], considering multilayer networks [15] [16] [17], considering the individual payoff weighting [18] and integrating the maximal neighbor’s payoff into the fitness in a network game [19]. In addition, several researchers have addressed an important issue that the payoffs stated in the payoff matrix of a game are not necessarily the only payoffs that a player perceives. Often, feelings like guilt and shame play a major role in decision-making in games as found by C. Boone et al [20], Matsumoto et al. [21] and Tabibnia [22], and the actions of players are influenced by emotions. One of the triggers that cause these feelings is that an opponent or the player herself deviates from a social norm [23] [24].

In this article we investigate the effects of including these emotions in the game setups. We extend some of the work of Roger Waldeck [25] where it is assumed that players abide by a commonly known norm and the feeling of *guilt* is introduced in a PD game. An agent experiences the feeling of guilt when she defects while her opponent cooperated. One could also imagine that the opposite could happen, i.e., the player cooperates but now the opponent deviates from the social norm to defect. This would cause the player to feel betrayed. We extend the work of Waldeck by introducing both feelings of guilt and betrayal in a general 2 × 2 two-player game. Based on the feelings the general game can become either a prisoner’s dilemma or a *snowdrift (SD)* game, two well-known games in the field of game theory [26]. We consider the snowdrift game because of its nice property that under the evolutionary dynamics determined by the replicator dynamics [27] and the best response update rule [28], the final population state is in general irrelevant to the initial population state in a group of agents playing the snowdrift game. Such a property is also observed in evolutionary prisoner’s dilemma games, but is less interesting since the final state corresponds to purely defectors. This independence of the initial population state in evolutionary PD and SD games helps us to scrutinize the effects of other factors such as the emotional feelings on the final population state without worrying about the effect of the initial population.

Since we take into consideration emotions, which typically differ for each individual, we use *asymmetric games* to better resemble real-life situations. Different setups have been suggested for studying heterogeneous populations [29] [30]. Moreover, the model in [25] already incorporates asymmetry by considering different levels of guilt among the individuals. We introduce asymmetry in a slightly different way but the concept remains the same. We assign the players with different ‘levels’ of emotions and thereby we create different types of players. We use an agent-based modeling approach to simulate the effects of adding different types of agents in a population. In the computations we assume that the agents in this framework have a *bounded rationality*, i.e., they get some information from the rest of the population and use this to ‘reason’ what would be the next best-move in the game.

We assign agents to have a pure strategy to either cooperate or defect. At each time-step, a randomly chosen agent gets to know the number of the agents who have cooperated (or defected) at the previous time-step, and then updates her strategy accordingly. An interpretation of such a population game is to randomly choose an agent at each time-step to play with all other agents, and then update her strategy accordingly. This interaction can be considered as a special case of the *round-robin tournament*[28] [31] where each agent plays with all other agents. It is assumed that the players in this context are *utility-maximizing* and therefore update their strategies based on their payoff matrices. They update according to the *myopic best response rule*[28], which means that each agent updates by choosing the best reply to the average population in the previous time-step. In other words, based on the last-played strategies in the population, an agent determines the probability of meeting a cooperator (including herself), and calculates her strategy accordingly. This is different from the *imitative update rule*[32] where each agent updates to the strategy of her neighbor with the highest payoff in the last (few) round(s).

## Framework

### A game with betrayal and guilt

In this article, we consider 2 × 2, asymmetric, two-player games. Each game involves a row-player and a column-player both of whom having the option to cooperate (C) or to defect (D).

Consider the following general form of 2 × 2 payoff matrices [1] for the row player:

$$\begin{array}{c} \pi =\begin{array}{cc} \; & \begin{array}{cc} C & D \end{array}\\ \begin{array}{c} C\\ D \end{array} & \left(\begin{array}{cc} R & S\\ T & P \end{array}\right) \end{array}. \end{array}$$ (1)

We call the payoff matrix a *Prisoner’s Dilemma (PD)* payoff matrix if the following condition holds

$$\begin{array}{c} T>R>P>S. \end{array}$$ (2)

The two inequalities *T* > *R* and *P* > *S* guarantee defection to be a better choice compared to cooperation. The inequality *R* > *P* is to assign mutual cooperation with a higher profit comparing to mutual defection. In addition to the PD case, we call the payoff matrix (Eq 1) a *Snow Drift (SD)* payoff matrix if the following condition holds

$$\begin{array}{c} T>R>S>P. \end{array}$$

Similarly, here the inequalities *T* > *R* and *S* > *P* determine the choice of strategy of the row player, which makes cooperation a better choice when the opponent defects and defection a better choice when the opponent cooperates; the inequality *R* > *S* advances mutual cooperation over mutual defection.

Another widely used notation for a 2 × 2 snowdrift payoff matrix, which we adopt throughout this paper, is

$$\begin{array}{c} \begin{array}{cc} \; & \begin{array}{cc} C\;\;\;\;\;\;\; & \;\;\;\;D \end{array}\\ \begin{array}{c} C\\ D \end{array} & \left(\begin{array}{cc} r-c/2 & r-c\\ r & 0 \end{array}\right) \end{array} \end{array}$$ (3)

where *r* is the *reward* for cooperation and *c* is the *cost* of cooperation. The payoffs of the above payoff matrix represent the *raw payoffs* of the row player [33] and are the “real,” non-emotional payoffs of the game, e.g., the money that one earns out of the game. We call the payoff matrix constructed of only raw payoffs, the *raw-payoff matrix*.

One can also include some *emotional payoffs* in the raw-payoff matrix. The emotional payoffs are those that are not officially stated but do play a major role in decision-making, e.g., the *feeling of guilt* one may have after defecting an opponent who has cooperated. We assume that there is a *social norm* that everybody should adhere to, which is to cooperate [34]. In [25], the feeling of guilt has been stated as an emotion that is perceived when an individual does not adhere to a social norm that all players agreed on. Because players are concerned with not only their own payoffs but also the (fair) distribution of payoffs for others, a player experiences guilt or shame for deviating from the social norm. We also call the feeling of guilt, the *guilt factor* or in short *guilt* and denote it by *g*.

In addition, what we add to this framework is another emotional payoff for the *feeling of being betrayed* which we also call the *betrayal factor* or in short the *betrayal* denoted by *b*. Feeling betrayed is an emotion that is experienced after the opponent breaches a socially accepted norm, i.e., your opponent violates your trust on him. Now since in the setting of this article, cooperation is set as the social norm, we state that a player experiences the feeling of being betrayed when her opponent deviates from the social norm by defecting while the player herself chose to cooperate. Note that here the values of *b*, *g*, *r* and *c* are considered to be positive unless otherwise mentioned.

We integrate the betrayal *b* and the guilt *g* into the raw-payoff matrix and obtain

$$\begin{array}{c} \begin{array}{cc} & \begin{array}{cc} C\;\;\;\;\;\;\;\;\;\;\;\; & \;D \end{array}\\ A=\begin{array}{c} C\\ D \end{array} & \left(\begin{array}{cc} r-c/2 & r-c-b\\ r-g & 0 \end{array}\right) \end{array}. \end{array}$$ (4)

To explain why *b* and *g* are positioned in the payoff matrix in the way that is stated above, we use the notion of *strategy profile* defined as the couple (*k*_*i*_, *k*_*j*_) where *k*_*i*_ and *k*_*j*_ are the pure strategies that the row-player *i* and column-player *j* have chosen, respectively. For example, when both players cooperate, the strategy profile is denoted as (*C*, *C*). We add the variables with a negative sign (since they have a negative effect on the payoffs) to the payoff matrix at the location that corresponds to the strategy profile that they are perceived. This means that −*g* is added to the bottom left corner which corresponds to the strategy profile (*D*, *C*) where the row-player defects and the column-player cooperates. On the other hand, −*b* is added to the top-right corner of the payoff matrix corresponding to the strategy profile (*C*, *D*), when the row-player cooperates and the column-player defects.

We set the following constraints on the entries of *A*:

$$\begin{array}{c} c>2g, \end{array}$$ (5)

$$\begin{array}{c} -2b<c<2r. \end{array}$$ (6)

Comparing *A* with the general-form payoff matrix *π*, one can easily see that inequality (Eq 5) satisfies the inequality *T* > *R* and (Eq 6) satisfies *R* > *S*, *P*. Now if the inequality *b* > *r* − *c* in *A* or correspondingly *P* > *S* in *π* holds, the payoff matrix becomes a PD payoff matrix. On the other hand, if the inequality *r* − *c* > *b* in *A* or correspondingly *S* > *P* in *π* holds, the payoff matrix becomes an SD payoff matrix. In other words, the magnitude of *b* with respect to *c* determines the type of *A*.

The payoffs in the payoff-matrix *A* are called the *all-in payoffs* of the row player which are her “all-things-considered” payoffs [33]. The all-in payoffs include officially stated payoffs, such as money, as well as the non-officially stated payoffs, such as the feeling of guilt. We call such a payoff matrix, i.e., constructed of all-in payoffs, the *all-in-payoff matrix*. According to the structure of the all-in payoff-matrix *A*, defection is no longer the best choice for a person with a great feeling of guilt, *g*, even when her opponent is cooperating and her raw-payoff matrix is a PD one. On the other hand, cooperation is not the best strategy for a person with a great feeling of being betrayed, *b*, even when her opponent is defecting and her corresponding raw-payoff matrix is an SD one. So in this sense, one can consider the all-in-payoff matrix to be a more “realistic” payoff matrix compared with the raw-payoff matrix.

### Types of agents

We consider *n* agents. Each agent *i* is assigned with a payoff matrix *A*_*i*_ of the same structure of *A* in (Eq 4); to be more precise, *A*_*i*_ is defined by

$$\begin{array}{c} \begin{array}{cc} & \begin{array}{cc} C\;\;\;\;\;\;\;\;\;\;\;\; & \;D \end{array}\\ A_{i}=\begin{array}{c} C\\ D \end{array} & \left(\begin{array}{cc} r-c/2 & r-c-b_{i}\\ r-g_{i} & 0 \end{array}\right) \end{array} \end{array}$$ (7)

where *g*_*i*_ and *b*_*i*_ are the guilt and betrayal of agent *i*, respectively and inequalities (Eq 5) and (Eq 6) hold when *b* and *g* are replaced by *b*_*i*_ and *g*_*i*_, respectively.

Let *n*_*l*_ be a non-negative integer. Based on the parameters of *A*_*i*_, each agent belongs to one of the following *n*_*l*_ + 1 types:

****PD** type:** The payoff matrix assigned to an agent *i* belonging to this type has the following property

$$\begin{array}{c} b_{i}>r-c. \end{array}$$

Comparing this payoff matrix with *π*, we know that the inequality *P* > *S* holds in this case. Hence, *A*_*i*_ is a PD payoff matrix in this case.

****SD**_*l*_ type** (*l* = 1,…, *n*_*l*_): The payoff matrix assigned to an agent *i* belonging to this type has the following properties:

$$\begin{array}{c} b_{i}=b_{SD_{l}},\;g_{i}=g_{SD_{l}},\;b_{SD_{l}}<r-c. \end{array}$$ (8)

Comparing this payoff matrix with *π*, we know that the inequality *S* > *P* holds in this case. Hence, *A*_*i*_ is an SD payoff matrix in this case.

We simply call a *PD*-type agent a *PD* agent, and an *SD*_*l*_-type agent an *SD*_*l*_ agent. In general, by an **SD* agent* we mean an agent belonging to one of the *SD*_*l*_ types. The type of an agent affects her choice of strategy against her opponent. As an illustration, for a *PD* agent it is always better to defect regardless of her opponent’s strategy.

### Strategy space and best-response

Denote the probability of a player *i* cooperating to be *x*_*i*_. Then the *mixed strategy* for player *i* which is the probability distribution over her set of pure strategies *C* and *D*, is defined by

$$\begin{array}{c} s_{i}=[\begin{array}{c} x_{i}\\ 1-x_{i} \end{array}]. \end{array}$$

The *utility function* of player *i* when playing against player *j* is defined by

$$\begin{array}{c} u_{i}\left(s_{i},s_{j}\right)={s_{i}}^{T}A_{i}\;s_{j} \end{array}$$ (9)

which according to (Eq 7) can be written into

$$\begin{array}{c} u_{i}\left(x_{i},x_{j}\right)=x_{i}(r-c-b_{i}+\left(b_{i}+\frac{c}{2}-r+g_{i}\right)x_{j})+x_{j}\left(r-g_{i}\right). \end{array}$$

Define $\beta_{i}^{*}(s_{j})$, the *pure-strategy best-reply correspondence* for player *i* to a strategy *s*_*j*_, as the set of pure strategies *k* ∈ {*C*, *D*} such that no other pure strategy gives her a higher payoff against *s*_*j*_ (this definition is in consistent with that in [35]):

$$\begin{array}{c} \beta_{i}^{*}\left(s_{j}\right)=\left\{k\in \left\{C,D\right\}:\;u_{i}\left(k,s_{j}\right)\geq u_{i}\left(x,s_{j}\right)\;\forall x\in \left\{C,D\right\}\right\} \end{array}$$

Note that there might be more than one best reply for player *i* to a strategy *s*_*j*_.

In order to calculate the pure-strategy best-reply correspondence, we have to find the pure strategy *k* such that *u*_*i*_(*k*, *s*_*j*_) is maximized. From the definition of *u*_*i*_(⋅,⋅), this is equivalent to player *i* choosing the maximum row of the multiplication *A*_*i*_ *s*_*j*_:

$$\begin{array}{c} A_{i}s_{j}=A_{i}[\begin{array}{c} x_{j}\\ 1-x_{j} \end{array}]=[\begin{array}{cc} r-c/2 & r-c-b_{i}\\ r-g_{i} & 0 \end{array}][\begin{array}{c} x_{j}\\ 1-x_{j} \end{array}]=[\begin{array}{c} \left(b_{i}+\frac{c}{2}\right)x_{j}+r-c-b_{i}\\ \left(r-g_{i}\right)x_{j} \end{array}]. \end{array}$$ (10)

Player *i* as a row player has to choose that pure strategy corresponding to the maximum entry of the final vector on the right-hand side in (Eq 10) in order to play her best response. Since Comparing the two entries of that vector $(b_{i}+\frac{c}{2})x_{j}+r-c-b_{i}$ and (*r* − *g*_*i*_)*x*_*j*_ is equivalent to compare $(b_{i}+\frac{c}{2}+g_{i}-r)x_{j}$ and *c* + *b*_*i*_ − *r*, we know that the pure-strategy best-reply correspondence of player *i* is

$$\begin{array}{c} \beta_{i}^{*}\left(s_{j}\right)=\{\begin{array}{cc} C & \left(b_{i}+\frac{c}{2}+g_{i}-r\right)x_{j}>c+b_{i}-r\\ C\;\text{or}\;D & \left(b_{i}+\frac{c}{2}+g_{i}-r\right)x_{j}=c+b_{i}-r\\ D & \left(b_{i}+\frac{c}{2}+g_{i}-r\right)x_{j}<c+b_{i}-r \end{array}. \end{array}$$

### Equilibrium point

Define the *equilibrium point of an *SD*_*l*_ agent* with the payoff matrix *A*_*i*_ in the general form of *π* in (Eq 1) to be

$$\begin{array}{c} x_{SD_{l}}^{*}=\frac{S-P}{T+S-R-P}. \end{array}$$ (11)

The equilibrium point has the property that an *SD*_*l*_ agent’s best response to an opponent with the cooperation probability lower than (resp. higher than) $x_{SD_{l}}^{*}$ is to cooperate (resp. defect). This property will be used later in the analysis.

**Remark 1** *The point* $x_{SD_{l}}^{*}$ *is the evolutionary stable strategy of the symmetric game with the snow-drift payoff matrix π. Moreover, the strategy profile* $\left(x_{SD_{l}}^{*},x_{SD_{l}}^{*}\right)$ *is the evolutionary stable equilibrium and hence the Nash equilibrium of such a game.*

### Population Game

Consider a population of *n* agents, each of whom belongs to one of the *n*_*l*_ + 1 types explained in subsection 1. For such a population, we define a *population game* as follows. The strategy of each agent is initialized to be either cooperation or defection. Then, at each time-step *t*, an agent *i* is randomly chosen from the whole population to update her strategy. The agent gets to know the ratio of cooperators in the population at *t* − 1, including herself in case her strategy was *C* at *t* − 1. This ratio is denoted by *x*_*C*_(*t* − 1) and is the same as the probability of a randomly chosen agent from the population at time *t* − 1 being a cooperator. Then agent *i* updates her strategy according to the *myopic best response (update) rule* [28] where a small randomness is added: With the probability 0.98, agent *i* updates her strategy to her pure-strategy best-reply correspondence against [*x*_*C*_(*t* − 1), 1 − *x*_*C*_(*t* − 1)]^*T*^, and the probability 0.02 she randomly chooses from cooperation and defection. In case her pure-strategy best-reply correspondence was either to cooperate or to defect, i.e., both *C* and *D* result in the same payoff, she will keep her pure strategy at time *t* − 1.

Let *p*_*i*_(*t*) denote the pure strategy of agent *i* at the time-step *t*. Also let Θ denote the 2-dimensional simplex, which is the convex combination of the two unit-vectors [1, 0]^*T*^ and [0, 1]^*T*^. Following the definition of best reply correspondence, we define the *i*th player’s *pure-strategy best-reply function* *β*_*i*_:Θ × ℝ → {*C*, *D*} at time *t* to be

$$\begin{array}{c} \beta_{i}\left(s_{j},t\right)=\{\begin{array}{cc} C & \left(b_{i}+\frac{c}{2}+g_{i}-r\right)x_{j}>c+b_{i}-r\\ p_{i}\left(t-1\right) & \left(b_{i}+\frac{c}{2}+g_{i}-r\right)x_{j}=c+b_{i}-r\\ D & \left(b_{i}+\frac{c}{2}+g_{i}-r\right)x_{j}<c+b_{i}-r \end{array}. \end{array}$$ (12)

Although for the case of $(b_{i}+\frac{c}{2}+g_{i}-r)x_{j}$ becoming equal to *c* + *b*_*i*_ − *r*, any strategy that player *i* chooses is a best response, we limit her to play her pure strategy at the previous time-step, *p*_*i*_(*t* − 1), in this case. The players in this context are confined to pure strategies; hence, we also call the pure-strategy best-reply function the *best reply* or *best response*.

Now the population game can be summarized as follows.

- Each agent is initially programed to a pure strategy: *C* or *D*.
- At each time-step *t*, an agent *i* is randomly chosen from the population to update her strategy.
- Agent *i* updates her strategy to $\beta_{i}(s_{C}(t-1),t)$ with the probability 0.98 and chooses a random strategy from the set {*C*, *D*} with the probability 0.02.

**Remark 2** *The fact that an agent i updates her strategy to β_i_(x_C_(t − 1), t) is equivalent to the situation when an agent i updates her strategy to the best reply against a randomly chosen agent from the population including herself. The strategy [x_C_(t − 1) 1 − x_C_(t − 1)]^T^ can be considered as the “average population’s strategy” at time t − 1. Hence, agent i is updating her strategy to the best response against the average population at the previous time-step.*

## Multiple types of agents in a population

After introducing the feeling of being betrayed and how it can be implemented in the payoff matrix of an agent, now we are interested in studying how the betrayal factor affects the evolution of the strategies of different agents in a population under the best-response update rule. In this section, a population of different types of agents having different betrayals, whose strategies evolve according to the best-response update rule, is investigated through simulations. The goal is to understand the effects of the betrayal factors assigned to the agents on the final level of cooperation in the population. We start with a population including only two different types and then, populations involving three types are taken into account. Lastly, we draw some conclusions for a setting where the population consists of a large, finite number of types of agents.

### Two types of agents

A population of 100 agents belonging to one of the two types *PD* or *SD*_1_ is studied. The following values are used for the parameters in the corresponding payoff matrices of the types of agents, *A*_*i*_s:

$$\begin{array}{c} r=5,\;c=1,\;g_{i}=0\;\forall i,\\ b_{i}=5\;\text{when}\;\text{agent}\;i\;\text{is}\;\text{a}\;PD\;\text{type},\\ b_{i}=3.5\;\text{when}\;\text{agent}\;i\;\text{is}\;\text{an}\;SD_{1}\;\text{type,}\;\text{i.e.,}\;b_{SD_{1}}=3.5. \end{array}$$

With these agents, we do simulations in MATLAB considering different compositions of the types. In each simulation, a fixed mixture of *PD* and *SD*_1_ agents play the population game explained in Subsection 1, for 1000 iterations. The initial number of cooperators is set to 20. Then the ratio of cooperators with respect to the number of iterations is plotted. To see the average outcome, an average of 20 simulations are depicted in the figures.

**Fig 1:**
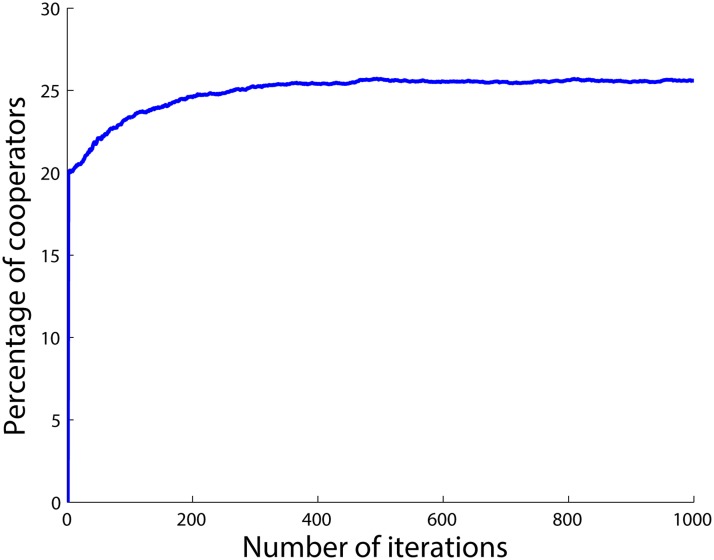
Percentage of cooperators when there are 75 *PD* and 25 *SD* agents.

The results when having 25 *SD*_1_ agents and 75 *PD* agents are shown in Fig 1. The figure shows that the total number of cooperators in the population quickly converges to the number of *SD*_1_ agents. In order to understand the behavior of the agents described above, we need to examine the best response of each type, which is done by studying the all-in-payoff matrix of the different types of agents. The all-in payoff matrices of the two types of agents are:

$$\begin{array}{c} \begin{array}{cc} & \begin{array}{cc} C\; & \;D \end{array}\\ A_{PD}=\begin{array}{c} C\\ D \end{array} & \left(\begin{array}{cc} 4.5 & -1\\ 5 & 0 \end{array}\right) \end{array}, \end{array}$$ (13)

$$\begin{array}{c} \begin{array}{cc} & \begin{array}{cc} C\;\;\; & D \end{array}\\ A_{SD_{1}}=\begin{array}{c} C\\ D \end{array} & \left(\begin{array}{cc} 4.5 & 0.5\\ 5 & 0 \end{array}\right) \end{array}. \end{array}$$ (14)

From the *PD* payoff matrix it can be seen that pure defection is the best option, i.e., no matter what the opponent’s strategy is, it is always best for the agent to defect. Correspondingly, one may say that defection is an equilibrium for *PD* agents. For the *SD*_1_ type payoff matrix, there is no pure equilibrium. Indeed, for *SD*_1_ agents it is most beneficial that one part of the population cooperates and the other part defects. To calculate how many cooperators and defectors are optimal for a particular *SD*_1_ agent, we use Eq (11) to derive the mixed strategy equilibrium point: $x_{SD_{1}}^{*}=\frac{S-P}{T+S-R-P}$. Replacing *T*, *S*, *R* and *P* by the values from the payoff matrix of the *SD*_1_ agents, *A*_*SD*_1__, results in $x_{SD_{1}}^{*}=0.5$. This implies that an *SD*_1_ agent “strives” for a population with 50% cooperators. In other words, an *SD*_1_ agent tries to modify her strategy in such a way that the ratio of cooperators in the population approaches this equilibrium point, $x_{SD_{1}}^{*}$. Therefore, an *SD*_1_ agent will defect when the ratio of cooperators in the population is greater than $x_{SD_{1}}^{*}$ and will cooperate when the ratio of cooperators has not yet reached the equilibrium point.

Now consider Fig 1 again. There are 75 *PD* agents in the corresponding population who always defect after a while, i.e., after when they have gotten the chance to update their initial strategies. For the *SD*_1_ agents on the other hand, it is most profitable to have a ratio of $x_{SD_{1}}^{*}=0.5$ cooperators in the whole population. In other words, *SD*_1_ agents aim to make the total number of cooperators in the population 50 and the total number of defectors also 50. However, the *PD* agents have already occupied 75 agents of the population, and they all defect. So there are only 25 spots left to cooperate. Hence, the best the *SD*_1_ agents can do is that all of them cooperate in order to get as close as possible to their ideal equilibrium point which happens with 50 cooperators. Hence, all *SD*_1_ agents will cooperate in the final state while all the *PD* agents defect. In other words, all the agents with the lower level of the betrayal factor (*SD*_1_s) cooperate and all the agents with the higher level of the betrayal factor (*PD*s) defect in the final state. The number of cooperators and defectors for each type of agents in the final state are provided in Table 1 where *n*_*C*_ and *n*_*D*_ are the number of cooperators and defectors, respectively.

**Table 1:** Agents’ strategies when there are 75 *PD* and 25 *SD*_1_ agents.

**Type**	**Number of cooperators**	**Number of defectors**	**Total number**
*PD*	0	75	75
*SD*	25	0	25
**Total**	25	75	100

**Fig 2:**
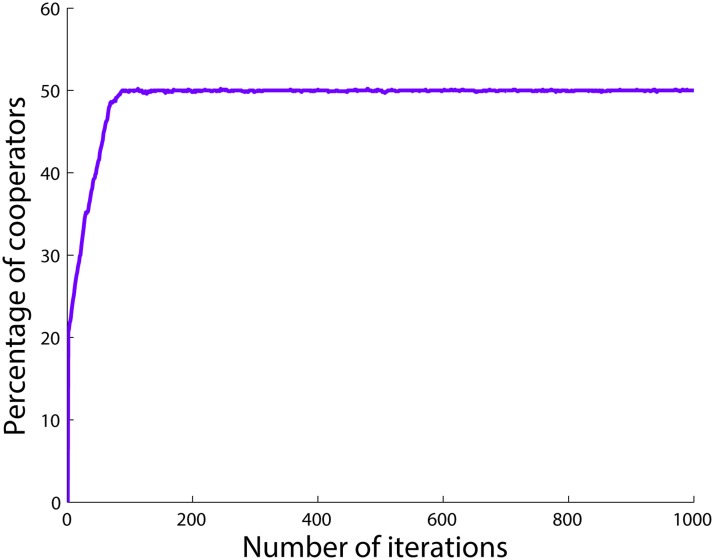
Percentage of cooperators when there are 25 *PD* and 75 *SD*_1_ agents.

One may guess that the number of the agents with the lower betrayal factor always determines the total number of cooperators in the final state of the population game. However, Fig 2 shows that such a conjecture is wrong. The figure shows the changes in the ratio of cooperators in the population when having 75 *SD*_1_ agents and 25 *PD* agents. As can be seen, the total number of cooperators in the population does not become equal to the number of *SD*_1_ agents in this case, but converges to a lower number, 50 (see Table 2). In this case, the number of *SD*_1_ agents, 75, is enough to provide the number of cooperators required for the equilibrium point $x_{SD_{1}}^{*}=0.5$. Hence, even though all the *PD* agents defect in the final state here as well as in the previous case, 50 of the *SD*_1_ agents cooperate, and the rest join the *PD* agents by defecting in order to make the total number of cooperators in the population equal to that of the equilibrium point $x_{SD_{1}}^{*}=0.5$. In this case, agents with a lower level of the betrayal factor are able to balance the strategies of the agents in the population in order to reach their desired number of cooperators. This is simply because the number of agents that feel less betrayed is greater than the required number of cooperators at the equilibrium point of these agents.

**Table 2:** Agents’ strategies when there are 25 *PD* and 75 *SD*_1_ agents.

**Type**	**Number of cooperators**	**Number of defectors**	**Total number**
*PD*	0	25	25
*SD*	50	25	75
**Total**	50	50	100

**Fig 3:**
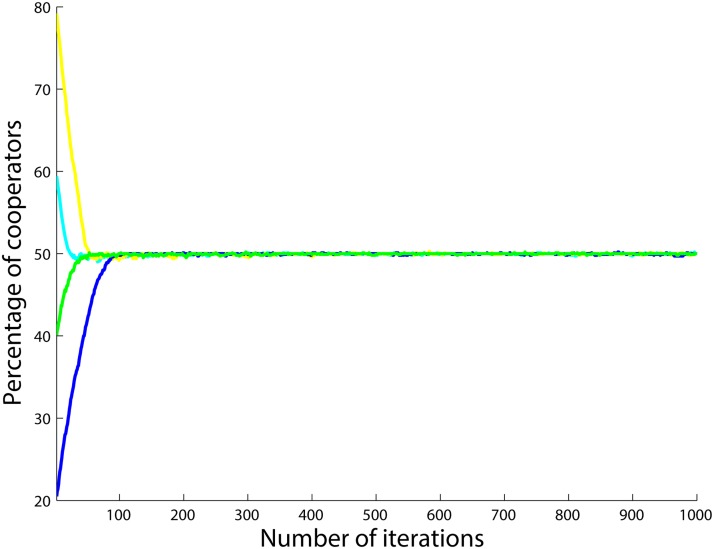
Percentage of cooperators when there are 25 *PD* and 75 *SD*_1_ agents. Four different initial numbers of cooperators are examined.

In order to show that the initial number of cooperators does not affect the final number of cooperators, we add Fig 3 where the population starts with different numbers of cooperators. As can be seen, no matter what the initial strategies of the agents are, and hence, how great the initial ratio of cooperators in the population is, the total number of cooperators in the population converges to the same value.

**Fig 4:**
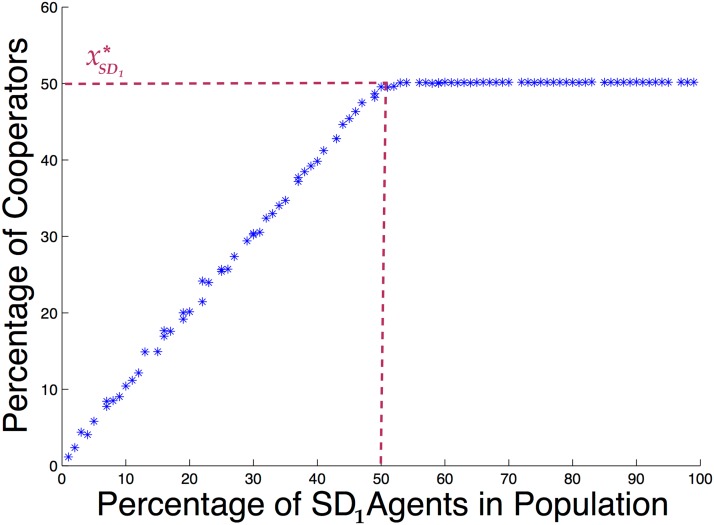
Percentage of cooperators when there are different portions of *SD*_1_ agents.

Now to further investigate how the number of agents having a lower value of betrayal factor, i.e., the *SD*_1_ types, affects the final level of cooperation in the population, we conduct 101 different simulations as follows: One simulation where all of the 100 agents are *PD* type, one where one agent is an *SD*_1_ type and the rest are *PD* type, one where two of the agents are *SD*_1_ and the rest are *PD*, and so on until where all of the 100 agents are *SD*_1_ type. In each simulation, the number of cooperators in the population is averaged from iteration number 700 to 1000. Consider this to be the *steady state* for this combination of *SD* and *PD* agents. The resulting steady states of each of these 101 simulations are depicted in Fig 4.

As can be seen, the ratio of cooperators starts from zero and grows constantly as the ratio of *SD*_1_ agents increases. At the equilibrium point of the *SD*_1_ agents, i.e., $x_{SD_{1}}^{*}=0.5$, this growth stops and the curve stays at a fixed percentage of cooperators while the number of *SD*_1_ agents continues to increase. The reason for this phenomenon lies in what we saw in the previous examples. Based upon the payoff matrix of the *PD* agents, we know full defection is always the best strategy for the *PD* agents. This implies that all of the *PD* agents will defect after they get the chance to update for the first time. This explains why the curve starts from the origin: When the population is completely made of *PD* agents, there will be no cooperators in the steady state. Moreover, because of the defection of all *PD* agents, the portion of cooperators in the population depends solely upon the portion of *SD*_1_ agents. Therefore, there is a linear relation in the graph between the portion of *SD*_1_ agents and the level of cooperation for portions of *SD*_1_ agents lower than $x_{SD_{1}}^{*}$. In other words, up to $x_{SD_{1}}^{*}$ the percentage of *SD*_1_ agents in the population is so low that it is beneficial for all of the *SD*_1_ agents to cooperate. Beyond this equilibrium, however, it is no longer beneficial for every *SD*_1_ agent to cooperate and some will start to defect so that the equilibrium point is maintained.

To conclude, in a population with two different types of agents there is a critical point for the number of agents with a lower betrayal factor. While the number of agents with the lower betrayal factor is smaller than that critical point, they all prefer to cooperate and raise the average level of cooperation. However, after the number of agents that feel less betrayed passes that critical point, their selfishness steps in and does not allow more cooperation to appear in the population.

### Three types of agents

Continuing with the previous subsection, we now add yet another type of agents to the population who again have an *SD* payoff matrix, but not exactly as the previous ones. This is done because we are interested in knowing how two conflicting equilibria of different *SD* agents will resolve. Adding another *PD* type of agents would not change much because the equilibrium of all *PD* agents is pure defection, which would be similar to adding more *PD* agents of the same type.

We run the simulations with the same settings as before. The same payoff matrices for the *PD* and *SD*_1_ agents are used and for the new type of agents, i.e., the *SD*_2_ agents, we set *b*_*i*_ = 2.5. The all-in-payoff matrix of every *SD*_2_ agent then becomes:

$$\begin{array}{c} \begin{array}{cc} & \begin{array}{cc} s_{j}=1 & s_{j}=0 \end{array}\\ P_{SD_{2}}=\begin{array}{c} s_{i}=1\\ s_{i}=0 \end{array} & \left(\begin{array}{cc} \;\;\;4.5\;\;\;\;\;\;\; & 1.5\;\;\;\;\\ 5\;\;\;\;\;\; & 0\;\; \end{array}\;\right) \end{array}. \end{array}$$ (15)

Based on this payoff matrix, the equilibrium point for an *SD*_2_ agent is $x_{SD_{2}}^{*}=0.75$ which is the desired ratio of cooperators in the population for the *SD*_2_ agents.

**Fig 5:**
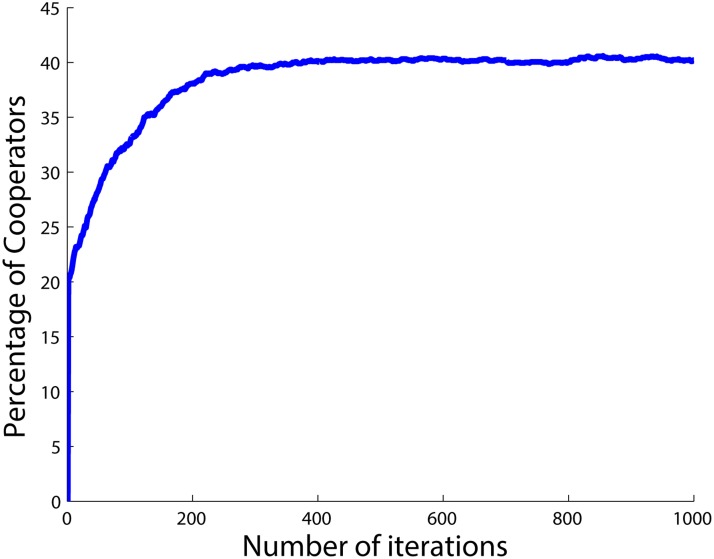
Percentage of cooperators when there are 60 *PD*, 20 *SD*_1_ and 20 *SD*_2_ agents.

We examine the behaviour of each of the types of agents in the population in a similar procedure to Section **Two types of agents**. Recall that for the *PD* and *SD*_1_ agents, the desired equilibria implied 0 and 50 cooperators respectively. Firstly, we consider a case where the population consists of 60 *PD*, 20 *SD*_1_ and 20 *SD*_2_ agents. The percentage of cooperators with respect to the time-steps is plotted in Fig 5. In this situation the *PD* agents will still be defecting. On the other hand, the *SD* agents will cooperate because the sum of both types of *SD* agents is lower than the lowest equilibrium of the two types of agents. This means that for both *SD* agents the equilibrium is not yet reached and the best strategy to approach this equilibrium is for them to cooperate. The results at the final state are summarized in Table 3.

**Table 3:** Agents’ strategies when there are 60 *PD*, 20 *SD*_1_ and 20 *SD*_2_ agents.

**Type**	**Number of cooperators**	**Number of defectors**	**Total number**
*PD*	0	60	60
*SD*_1_	20	0	20
*SD*_2_	20	0	20
**Total**	40	60	100

The situation changes when the total number of potential cooperators in the population, i.e., *SD* agents, rises above 50 which is the equilibrium point of the *SD*_1_ agents. Then, some of the *SD*_1_ agents, who have the medium level of the betrayal factor among the three available types of agents in the population will start to defect to maintain their equilibrium, $x_{SD_{1}}^{*}=0.50$, as mentioned before. We summarize the behaviour of the agents in Table 4, where we consider a population with 40 *PD*, 20 *SD*_1_ and 40 *SD*_2_ agents.

**Table 4:** Agents’ strategies when there are 40 *PD*, 20 *SD*_1_ and 40 *SD*_2_ agents.

**Type**	**Number of cooperators**	**Number of defectors**	**Total number**
*PD*	0	40	40
*SD*_1_	10	10	20
*SD*_2_	40	0	40
**Total**	50	50	100

In Table 4, one can see that 10 *SD*_1_ agents defect to maintain the equilibrium of 50 cooperators. All of the *SD*_2_ agents, who have the lowest level of the betrayal factor, cooperate because they want the population to reach 75 cooperators in the population.

This process is continued until all of the *SD*_1_ agents defect and they can no longer maintain their equilibrium. In other words, if the number of *SD*_2_ agents becomes more than 50, also the ratio of cooperators in the population will grow beyond that of $x_{SD_{1}}^{*}=0.50$ as can be seen in Table 5.

**Table 5:** Agents’ strategies when there are 29 *PD*, 20 *SD*_1_ and 51 *SD*_2_ agents.

**Type**	**Number of cooperators**	**Number of defectors**	**Total number**
*PD*	0	29	29
*SD*_1_	0	20	20
*SD*_2_	51	0	51
**Total**	51	49	100

**Fig 6:**
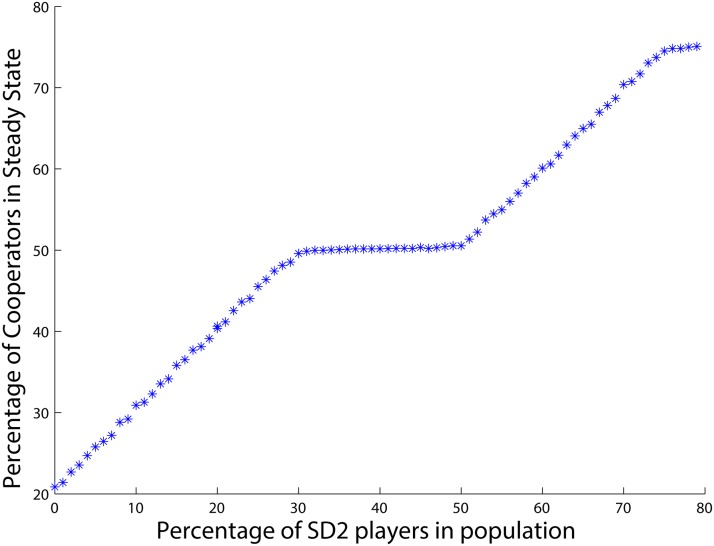
Percentage of cooperators when there are 20 *SD*_1_ and varying *PD* and *SD*_2_ agents.

As for the case of two types of agents we can plot all of the steady states for the different combinations of *PD*, *SD*_1_ and *SD*_2_ agents in one figure. For example, Fig 6 shows the steady states of the percentage of cooperators for different combinations of *SD*_2_ and *PD* agents who have the lowest and highest levels of the feeling of being betrayed, while having the number of *SD*_1_ agents fixed to 20. The figure includes the cases of the previous tables. As can be seen, in the absence of the *SD*_2_ agents, all *SD*_1_ agents, who now are the type with the least betrayal factor in the population, cooperate in the final state. That is why the curve starts from 20 percentage cooperation in the steady state. By the introduction of some even more cooperative type of agents, i.e., *SD*_2_s, the total number of cooperators in the long run increases, but only up to some level where the selfishness of the agents with the medium feeling of being betrayed, i.e., *SD*_1_s, rises and tries to maintain the level of cooperation at their own desired level. However, after a while, by further increasing the number of the agents that have the least feeling of being betrayed, i.e., *SD*_2_s, the agents with the medium level of the betrayal factor, i.e., *SD*_1_s, can no longer balance the total number of cooperators. Then the average cooperation level in the population rises by the increments in the number of the agents with the lowest feeling of being betrayed, but limits off at the point where the selfishness of this even very unselfish group shows up.

**Fig 7:**
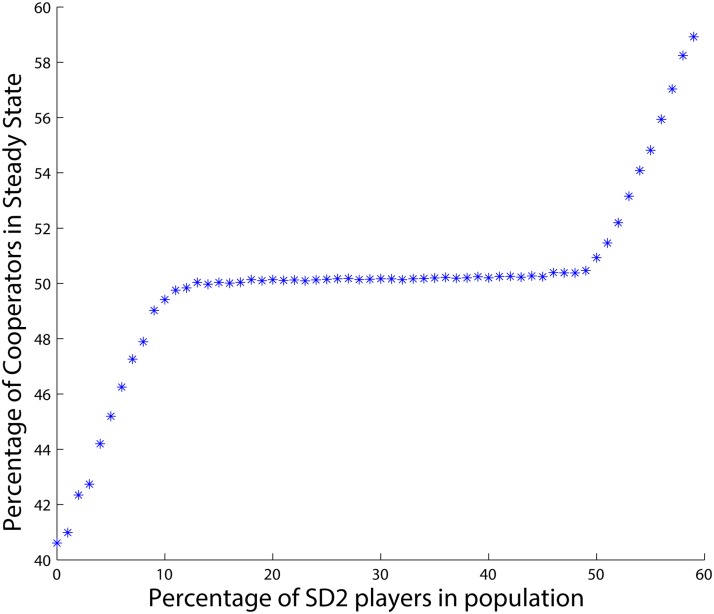
Percentage of cooperators when there are 40 *SD*_1_ and varying *PD* and *SD*_2_ agents.

Another scenario is plotted in Fig 7 where there are 40 *SD*_1_ agents and the number of *PD* and *SD*_2_ agents vary in the *x*-axis. As can be seen, the number of *SD*_1_ agents, i.e., the number of the agents with the medium betrayal factor, is so big that they do not allow the most cooperative type that has the lowest level of the feeling of being betrayed, i.e., *SD*_2_, to reach its equilibrium even when *SD*_2_ agents have their highest possible portion of the population.

#### A comprehensive 3D plot of the results

**Fig 8:**
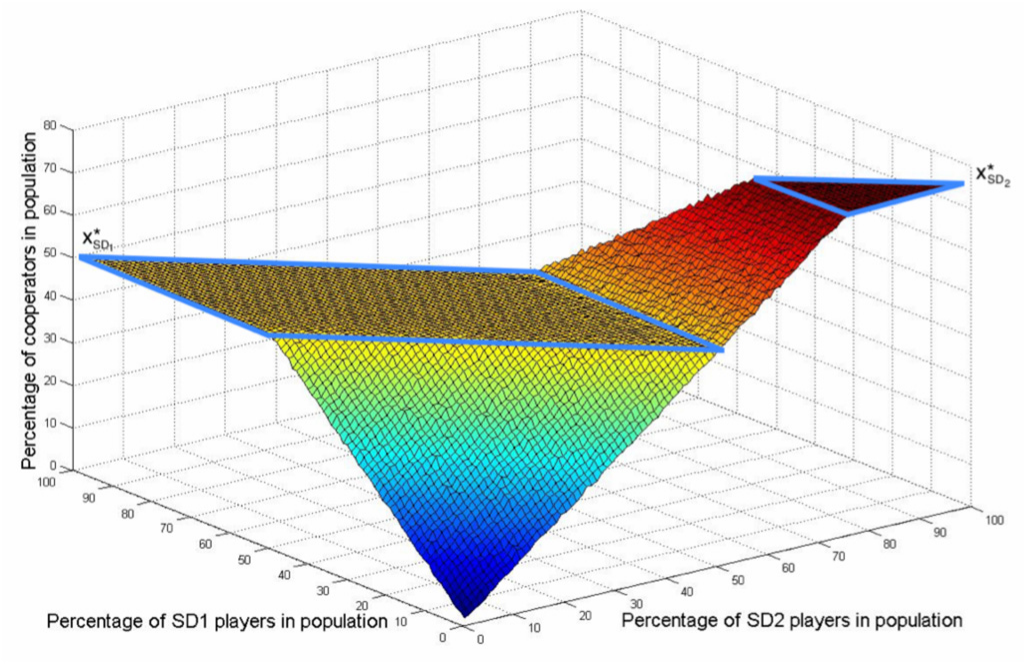
Percentage of cooperators when there are different portions of *SD*_1_ and *SD*_2_ agents.

One may plot the steady states of the percentage of cooperators for all different combinations of *PD*, *SD*_1_ and *SD*_2_ agents in one figure. This results in the 3D plotting of Fig 8. Each point in Fig 8 is a representation of the steady state of a population with a certain number of *PD*, *SD*_1_ and *SD*_2_ agents. Note that the curves in Fig 7 and Fig 6 are two vertical slices of this 3D figure. One can distinguish two horizontal areas and two sloped areas in Fig 8. In the large horizontal area of the figure, marked with blue lines and denoted by $x_{SD_{1}}^{*}$, all of the steady states are equal to the equilibrium of the *SD*_1_ agents. There, the *SD*_1_ agents have been able to maintain their desired level of cooperators by modifying their strategy accordingly. The same holds for the smaller horizontal area denoted by $x_{SD_{2}}^{*}$ where the *SD*_2_ agents have maintained their equilibrium.

In the sloped areas, the steady state of the population lies between two equilibria. In the main sloped area, coloured blue to yellow, the number of *SD* agents is relatively low and therefore the steady states lay between $x_{PD}^{*}=0$ and $x_{SD_{1}}^{*}=0.5$. The points in the sloped area in red obviously lay between the equilibrium points of both of the SD agents, $x_{SD_{1}}^{*}=0.5$ and $x_{SD_{2}}^{*}=0.75$.

We can explain the occurrence of these flat areas in the figure by looking at the equilibrium points of each of these types of agents. Each type of agent has a certain equilibrium point in terms of the ratio of cooperators in the population. For the *PD* agents this is zero, as mentioned before, because it is always most beneficial to defect according to their payoff matrices. Naturally, a *PD* agent which carries the highest level of the feeling of being betrayed among the agents, receives the greatest payoff when defecting while her opponent cooperates. This behaviour is called *free-riding*: Enjoying the contributions of others while not paying any of the costs. This is not the case for *SD* agents who have lower levels of the feeling of being betrayed. They receive the highest payoffs when the portion of cooperators in the population is optimally divided in two parts for this particular type of *SD* agents. Consequently, in a population with different types of *SD* agents these equilibria are conflicting. Each agent will strive to maintain or approach its own equilibrium point by modifying her strategy in such a way that the portion of cooperators approaches her equilibrium point which is of course determined by her betrayal factor.

For example, with the current settings, the *SD*_1_ agents will strive for an equilibrium of 50 cooperators. Up to this equilibrium, each *SD*_1_ agent will cooperate, as well as the *SD*_2_ agents, whose equilibrium point is even greater. This explains the main sloped-area, colored blue to yellow. However, above this point of 50 cooperators, the *SD*_1_ agents will start to defect to ‘neutralise’ the behaviour of others and maintain this equilibrium point. This illustrates the rectangular-shaped horizontal area. *SD*_2_ agents who feel the lowest level of being betrayed, will still be cooperating because they want an equilibrium with a higher ratio of cooperators. This continues until all of the *SD*_1_ agents defect and all *SD*_2_ agents cooperate. Then the number of *SD*_2_ agents is enough to reach the number of cooperators at their equilibrium point $x_{SD_{2}}^{*}$. This explains the sloped area in red. Afterwards, the selfishness of the *SD*_2_ agents forces them to balance the number of cooperators and defectors in the population to maintain their equilibrium while all *PD* and *SD*_1_ agents are defecting. This explains the triangle-shaped horizontal area on the top.

In short, based on the simulations, increasing the number of low-betrayal-level agents does not necessarily increase the average cooperation level in the population. Indeed, it depends on how the betrayal factor is distributed among the agents. For example, if the number of low-betrayal-level agents is big enough so that the average level of cooperation is already affected by these agents, then increasing the number of low-betrayal-level agents almost always increases the average cooperation level. On the other hand, if the number of low-betrayal-level agents is small compared to that of the high-betrayal-level agents so that the average level of cooperation is totally determined by high-betrayal-level agents, then almost always, increasing the number of low-betrayal-level agents does not change the average cooperation level. The reason is that high-betrayal-level agents will compensate this addition by defecting. However, this resistance of the high-betrayal-level agents is only up to some level. In other words, if we keep increasing the number of low-betrayal-level agents, after some point, finally the cooperation level starts to increase. This is because even though all of the high-betrayal-level agents will defect, their population size is limited and cannot compensate a large number of cooperators in the population.

### Large number of different types of agents

After simulating with 2 and 3 different types of agents in a population, we find that different equilibria can be attained in a population with multiple types of agents by changing the number of agents of a certain type. We extend this work by studying a population with a large but finite number of different types by using a *normal distribution* of the betrayal factor *b*_*i*_ over the agents. Different mean (*μ*) and variance (*σ*) values are used for this normal distribution to see the impact of these factors.

In general, to be able to predict the number of cooperators in the steady state, one can use the previous analysis of the behaviour of different types of agents:

- All of the agents want to reach and maintain their equilibrium by adapting their strategy accordingly.
- All of the *PD* agents always defect in the steady state.

From the previous simulations we know that the number of cooperators in the steady state is mainly determined by the equilibrium point of a certain type of agents. We use the average equilibrium point of all of the agents in the population to predict the ratio of cooperators in the final state. The results of the simulations with a normally distributed *b*_*i*_ are compared with the values of the average equilibrium and are stated in Table 6.

**Table 6:** Results for 100 different types of agents for several settings with normally distributed *b*_*i*_.

**a**	***μ***	***σ***	**average b**	**long-term ratio of cooperators from simulation**	**long-term ratio of cooperators from calculation**	**difference**
1	5	1	1,4	0,8429	0,838709677	-0,004190323
1	5	3	1,133	0,8522	0,851499851	-0,000700149
1	5	5	1,08	0,8521	0,85380117	0,00170117
2	5	1	2,4	0,7814	0,761904762	-0,019495238
2	5	3	2,133	0,7923	0,788762146	-0,003537854
2	5	5	2,08	0,7922	0,79338843	0,00118843
3	5	1	3,4	0,6452	0,545454545	-0,099745455
3	5	3	3,144	0,6417	0,631268437	-0,010431563
3	5	5	3,08	0,6521	0,647887324	-0,004212676
4	5	1	4,4	0,0072	-4	-4,0072
4	5	3	4,133	0,008	-0,36239782	-0,37039782
4	5	5	4,08	0,0141	-0,19047619	-0,20457619
5	5	1	5,4	0,0058	1,555555556	1,549755556
5	5	3	5,133	0,0067	1,789889415	1,783189415
5	5	5	5,08	0,0083	1,862068966	1,853768966
10	5	1	10,4	0,011	1,084745763	1,073745763

From the table one can conclude that the ratio of cooperators in the population at the steady state tends to the equilibrium of the “average” type of agents. Furthermore, the results show that the bigger the variance and therefore the less clear the average agent type, the more difference there is between the expected number of cooperators in the steady state and the realized steady state situation. This implies that if there is a clear majority in the population, then the overall number of cooperators would tend to the equilibrium point of that majority.

To test this hypothesis, we repeated the simulation with a finite number of different agent types with *b*_*i*_ being *uniformly distributed*. The motivation is that there is still a clear average; however, there are as many agents with an ‘extreme’ value as there are around the average. Therefore, there is no majority that can determine the number of cooperators in the steady state. The results of these experiments (see Table 7) show that for populations with no clear majority of agents of a certain type, the current method does not provide an appropriate approximation of the number of cooperators in the steady state.

**Table 7:** Results for 100 different types of agents for several settings with uniformly distributed *b*_*i*_.

**min**	**max**	**avg**	**long-term ratio of cooperators from simulation**	**long-term ratio of cooperators from calculation**	**difference**
0,1	1,1	0,6	0,8589	0,871794872	0,012894872
0,1	2,1	1,1	0,8188	0,852941176	0,034141176
0,1	3,1	1,6	0,7615	0,827586207	0,066086207
0,1	4,1	2,1	0,6959	0,791666667	0,095766667
0,1	5,1	2,6	0,6184	0,736842105	0,118442105
0,1	6,1	3,1	0,5542	0,642857143	0,088657143
0,1	7,1	3,6	0,4884	0,444444444	-0,043955556
0,1	8,1	4,1	0,4408	-0,25	-0,6908
0,1	9,1	4,6	0,4029	6	5,5971
0,1	10,1	5,1	0,3715	1,833333333	1,461833333

### Expected ratio of cooperators in the stationary state

It is also possible to estimate the ratio of cooperators in the steady state using the distribution of the betrayal factor in the population. We aim to find the equilibrium point of the population game explained in subsection 1 when the distribution of *b*_*i*_ over the agents is known and the rest of the parameters in the payoff matrix *A*_*i*_ are the same for all of the agents. We neglect the 0.02 chance that the randomly chosen agent updates to a random strategy. Define the function *a*:[0,1) → ℝ to be

$$\begin{array}{c} a\left(x\right)=\frac{(\frac{c}{2}+g_{i}-r)x+r-c}{1-x}. \end{array}$$

Then according to (Eq 12), the best response function can be written as

$$\begin{array}{c} \beta_{i}\left(s_{j},t\right)=\{\begin{array}{cc} C & b_{i}<a\left(c_{j}\right)\\ p_{i}\left(t-1\right) & b_{i}=a\left(c_{j}\right)\\ D & b_{i}>a\left(c_{j}\right) \end{array}. \end{array}$$ (16)

Based on the above equality, the probability of some agent *i* cooperating, *x*_*i*_, equals the probability of *b*_*i*_ being less than *a*(*x*_*j*_) when *x*_*j*_ ≠ 1:

$$\begin{array}{c} x_{i}=\mathrm{Pr}(b_{i}<a\left(x_{j}\right)). \end{array}$$ (17)

When *x*_*j*_ = 1, we have that *x*_*i*_ = 0 which is the case when one player cooperates and the other defects. Now let *F*_*b*_(⋅) denote the *cumulative distribution function* of the agents’ betrayal factors. Then Eq (17) can be written as

$$\begin{array}{c} x_{i}=F_{b}(a(x_{j})). \end{array}$$ (18)

Now consider the population game in the stationary state, i.e., when an agent no longer changes her strategy when being chosen to update. Denote the expected proportion of cooperators in the stationary state by *x*_*C*_. By definition, *x*_*C*_ equals the probability that in the stationary state, the strategy of a randomly chosen agent is *C*. Since the agents are at the stationary state, *x*_*C*_ equals the probability that a randomly chosen agent cooperates. On the other hand, according to (Eq 18), the probability of a random agent cooperating equals the probability of her betrayal being less than *a*(*x*_*j*_) when *x*_*j*_ ≠ 1. Note that *x*_*j*_ is the chance that the opponent cooperates; however, the opponent is also randomly chosen (which may be the agent herself). Hence, *x*_*j*_ = *x*_*C*_. Therefore, (Eq 18) becomes

$$\begin{array}{c} x_{C}=F_{b}(a(x_{C})). \end{array}$$ (19)

When *x*_*j*_ = 1, we have that *x*_*C*_ = 1 implying that all of the agents in the population are cooperators, which almost never happens since the steady state for the *PD* and *SD* types is to have all and some portion of the population to defect, respectively. So in general, the ratio of cooperators in the population game in the steady state can be approximated by (Eq 19).

## Varying emotions

Up to now, we have considered a simplified version of emotions by including a fixed number, the betrayal factor, in the payoff matrix of the agents. In reality, emotions fluctuate over time. In experiments with social dilemmas such as the *PD* game and other public goods games, it is observed [36] that people often start out cooperative but after a few disappointments will start to behave less cooperatively. This gives rise to the suggestion that the factor *b* should not be fixed but a function of the number of encounters with a defector, namely the *disappointments*.

In order to model such a varying feeling of being betrayed, we define a *varying emotion game* similar to the population game defined previously in Subsection 1 as follows. Consider a population of *n* agents, to each of which the payoff matrix *A*_*i*_ defined in (Eq 7) fulfilling the conditions (Eq 5) and (Eq 6) is assigned. Moreover, agents meet and play a game to be explained later. We assume that each agent has a memory that can store the number of times she has encountered a cooperator, *n*_*C*_, and the number of times she has encountered a defector, *n*_*D*_. The varying emotion game for such a population is defined to be the same as a population game with the following extra steps:

- After the chosen agent *i* updated her strategy, another agent *j* is randomly chosen from the population.
- Agent *i* as a row player plays a 2 × 2 asymmetric game against agent *j* as the column player where the payoff matrices *A*_*i*_ and $A_{j}^{T}$ correspond to the column and row players, respectively.
- Agent *i* updates her betrayal factor using a function *b*:ℝ^2^ → ℝ as
  $$\begin{array}{c} b_{i}=b\left(n_{C},n_{D}\right). \end{array}$$

Note that the betrayal factor *b*_*i*_ affects the agent’s benefits perceived from cooperation and her willingness to cooperate. Consequently, the agent chooses to update based on the probability that she will meet a cooperator next time and her willingness to cooperate for the next round.

**Fig 9:**
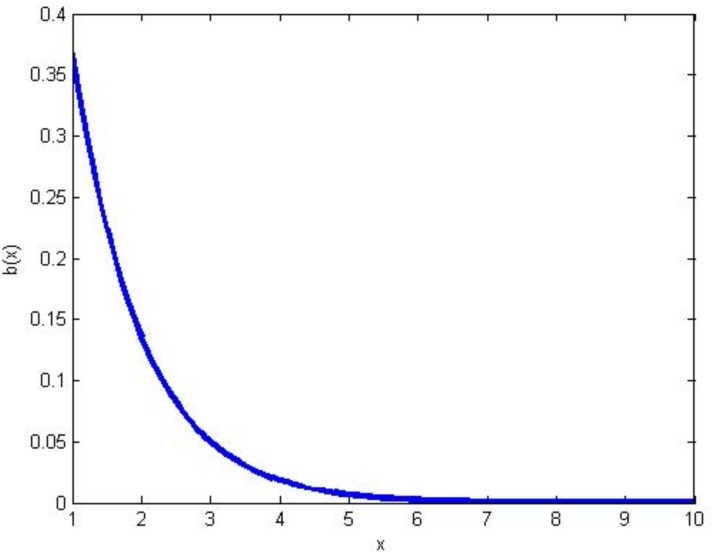
The exponential decay of b(1,1).

One of the simplest functions one can imagine for *b* is a linear function of *n*_*D*_:

$$\begin{array}{c} b\left(n_{C},n_{D}\right)=a_{1}-a_{2}n_{D} \end{array}$$

for some real parameter *a*_1_ and a positive *a*_2_. In this case, the betrayal would be a strictly decreasing function, which means that the probability that the corresponding agent will cooperate after many encounters with defectors tends to zero. One problem with such a function is that the betrayal of the agent gets affected equally after meeting a defector for the first time or for several times. However, in ‘reality’ it is more probable that the first negative encounter really touches an agent’s feelings, but after a while, the agent numbs and gets used to being defected upon and will start to defect as well. So it would be more realistic to choose a negative exponential function for *b*:

$$\begin{array}{c} b\left(n_{C},n_{D}\right)=a_{1}e^{-a_{2}n_{D}} \end{array}$$

where *a*_1_ and *a*_2_ are positive. Now the first encounters with a defector have a large negative effect; however, as *n*_*D*_ increases further, that is to meet more defectors, the magnitude of *b* slowly tends to zero. The exponential curve of *b* for parameters *a*_1_ and *a*_2_ set to 1 is depicted in Fig 9. The main problem with such an exponential function is that no matter how many times an agent encounters a cooperator, her betrayal increases, and hence, her willingness to cooperate decreases by increments in meeting defectors. Based on the fact that someone’s willingness to cooperate can both grow and diminish over time, we modify the above function to capture the effect of meeting cooperators as in the following

$$\begin{array}{c} b\left(n_{C},n_{D}\right)=a_{1}e^{-\frac{a_{2}n_{D}}{n_{C}+n_{D}}+\frac{a_{3}n_{C}}{n_{C}+n_{D}}}. \end{array}$$ (20)

As emotions fade over time and are put in perspective as an agent gains experience, we moderate the emotions by the total number of games that the agent has played, *n*_*C*_ + *n*_*D*_. The effect of each of the encounters can be enlarged or diminished by the variables *a*_2_ and *a*_3_.

**Fig 10:**
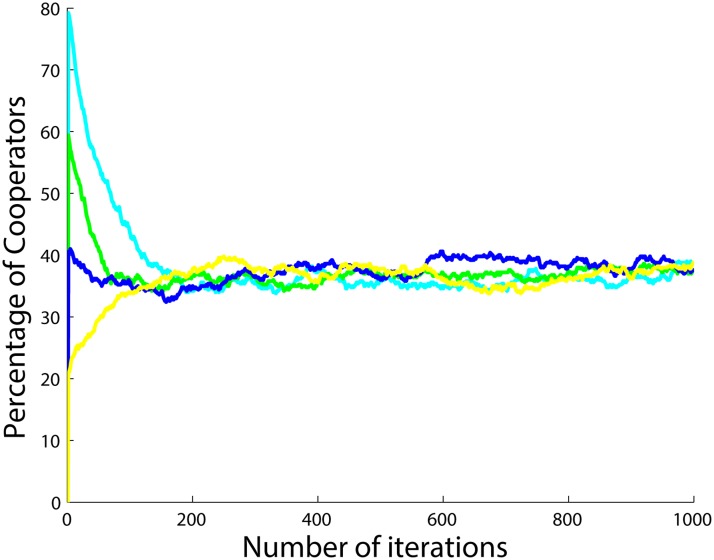
Percentage of cooperators with respect to time for 4 different initial conditions for 5 runs and 1000 iterations per simulation with the initial number of cooperators set to 20 (yellow), 40 (blue), 60 (green) and 80 (cyan) with *a*_1_ = *a*_2_ = *a*_3_ = 1 and when *r* = 5, *c* = 1 and *g*_*i*_ = 0.

**Fig 11:**
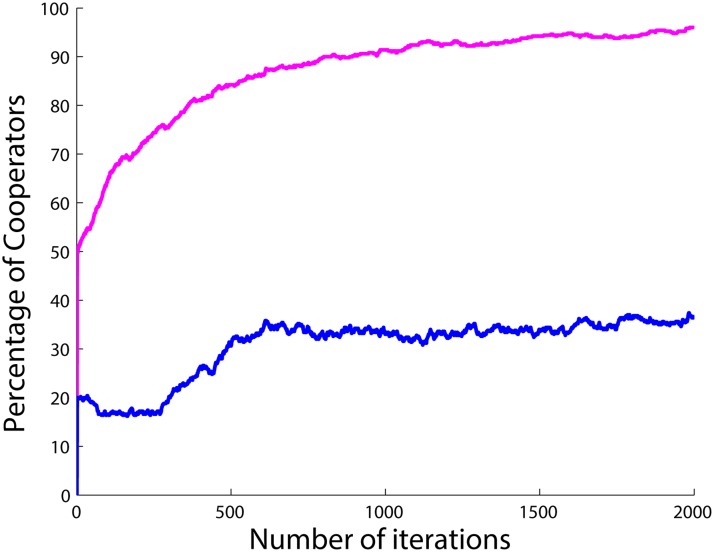
Percentage of cooperators with respect to time for 2 different initial conditions for 5 runs and 2000 iterations per simulation with the initial number of cooperators set to 20 (blue) and 50 (magenta) with *a*_1_ = 100 and *a*_2_, *a*_3_ = 1 and when *r* = 5, *c* = 1 and *g*_*i*_ = 0.

Now, with such a varying betrayal for each of the agents, we simulate the varying emotion game for a population of 100 agents, with the parameters *r* = 5, *c* = 1 and *g*_*i*_ = 0 in the payoff matrix *A*_*i*_ and for all *i*. The evolution of the percentage of cooperators for different initial number of cooperators is shown in Figs 10 and 11.

As can be seen from Fig 10, for the case of *a*_1_ = *a*_2_ = *a*_3_ = 1, for different initial numbers of cooperators in the population, the number of cooperators converges to approximately 40 in the steady state. However, for the case of *a*_1_ = 100 and *a*_2_, *a*_3_ = 1, there is a strong influence of the initial conditions as can be seen in Fig 11. For values greater than or equal to 50 percent initial cooperators, the population almost always converges to full cooperation in the long run. For values smaller than 50 percent initial cooperators, the number of cooperators converges to the same value 40 as before.

### Emotion-based decision-making

Until now, the assumption was made that all of the players know what percentage of the population has played cooperation in the previous round. This could be a reasonable assumption in a controlled game setting or in small groups where the behaviour of everybody is visible to everyone. However, in large groups it is unlikely that every player can make a good estimation of the probability of meeting a cooperator in the next round. Therefore, to be thorough, also some simulations are made where agents only update their strategies based upon their willingness to cooperate or put differently, on the amount of cooperators and defectors they met during the course of the game. The agents solely base their strategies, i.e., to cooperate or to defect, upon whether their willingness to cooperate is above or below a certain fixed level, 1 in this case, respectively. The willingness to cooperate is determined by the negative of the function *b* in (Eq 20). Hence, each agent cooperates if her *b* is greater than 1 and defects if her *b* is less than 1.

**Fig 12:**
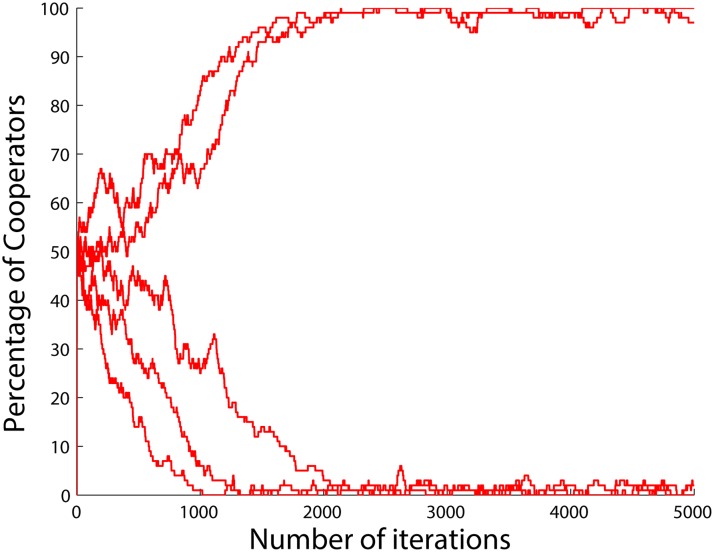
Different outcomes for the same initial conditions when the betrayal factors of the agents change by the function *b*(.) in (Eq 20) and also when the agents do not update their strategies, but instead cooperate when *b* < 1 and defect when *b* > 1. *a*_1_, *a*_2_ and *a*_3_ are all set to 1.

For the simulations, first all of the variables *a*_1_, *a*_2_ and *a*_3_ are kept to 1 and the strategies of half of the agents which are randomly chosen are initially set to cooperation, and the rest to defection. Note that *a*_1_ = *a*_2_ implies that the absolute size of the effect of meeting a cooperator or a defector are considered to be equal for each agent. The corresponding results can be found in Fig 12.

As can be seen, for the same initial number of cooperators, two completely different outcomes can happen in the final state: *Full defection*, i.e., a population of all defectors or *full cooperation*, i.e., a population of all cooperators. This implies that the sequence of the chosen agents to update their strategies plays an important role in this case. When the number of initial cooperators is higher than 50, the steady state almost always tends to full cooperation and vice versa.

**Fig 13:**
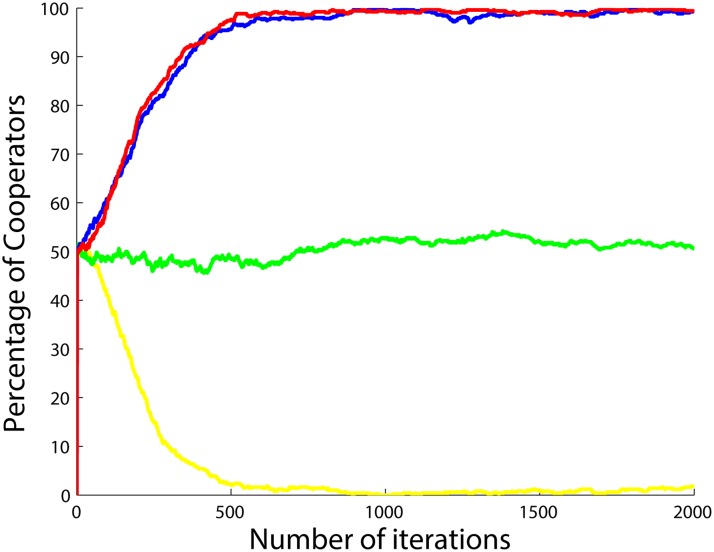
Percentage of cooperators with respect to the number of iterations when the betrayal factors of the agents change according to the function *b*(.) in (Eq 20) and also when the agents do not update their strategies, but instead cooperate when *b* < 1 and defect when *b* > 1. Four different situations are shown after 5 runs and 2000 iterations per simulation with *a*_1_ set to 0.5 (yellow), 1 (green), 1.5 (blue) and 2 (red), *a*_2_ and *a*_3_ both set to 1.

**Fig 14:**
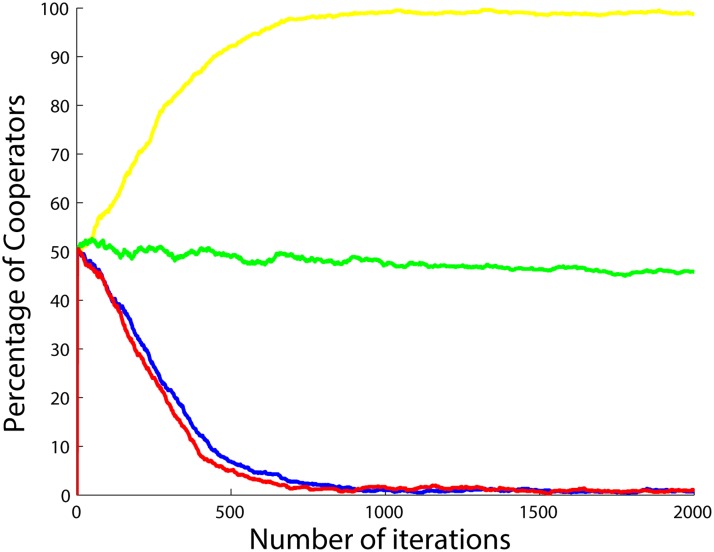
Percentage of cooperators with respect to the number of iterations when the betrayal factors of the agents change by the function *b*(.) in (Eq 20) and also when the agents do not update their strategies, but instead cooperate when *b* < 1 and defect when *b* > 1. Four different situations are shown after 5 runs and 2000 iterations per simulation with *a*_2_ set to 0.5 (yellow), 1 (green), 1.5 (blue) and 2 (red), *a*_1_ and *a*_3_ both set to 1.

**Fig 15:**
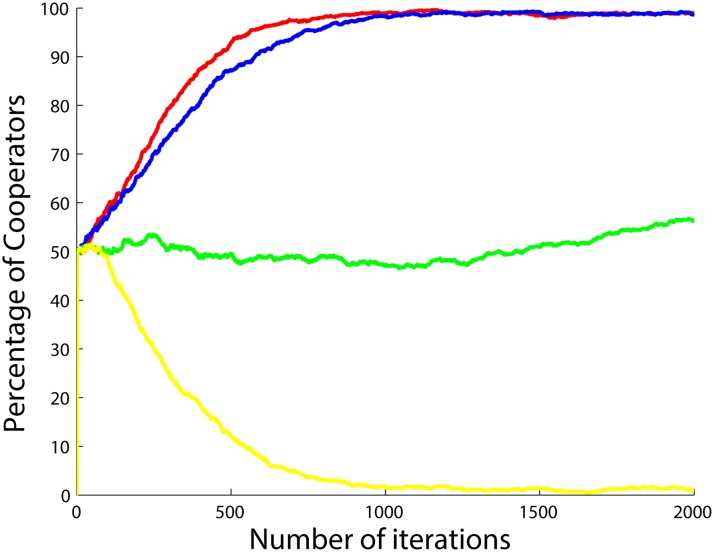
Percentage of cooperators with respect to the number of iterations when the betrayal factors of the agents change by the function *b*(.) in (Eq 20) and also when the agents do not update their strategies, but instead cooperate when *b* < 1 and defect when *b* > 1. Four different situations are shown after 5 runs and 2000 iterations per simulation with *a*_3_ set to 0.5 (yellow), 1 (green), 1.5 (blue) and 2 (red), *a*_1_ and *a*_2_ both set to 1.

It is also possible to turn the population to full defection or full cooperation almost independent of the initial number of cooperators by tuning the parameters *a*_1_, *a*_2_ and *a*_3_. In Figs 13–15, each of the variables *a*_1_, *a*_2_ and *a*_3_ are varied in turn to see what their respective effects on the levels of cooperation are. From the figures one can see that large values of *a*_1_ and *a*_3_ will lead to full cooperation and larger values of *a*_2_ will lead to full defection. This knowledge can be used to tune the model once experimental data from social experiments are known.

## Concluding remarks

The results of this paper help to understand the behavior of a population of individuals with different emotions participating in asymmetric games. We introduce a payoff for the feeling of being betrayed and incorporate it with another emotional payoff, guilt, in a 2 × 2 payoff matrix. According to the emotions of each agent, such a payoff matrix is assigned to her which has the structure of the payoff matrix of either a snowdrift or a prisoner’s dilemma game. For large values of the feeling of being betrayed, the payoff matrix takes the structure of a PD payoff matrix while for smaller values it can take the structure of an SD payoff matrix. This makes defection the best response for an agent having a great feeling of being betrayed while for others it depends on the strategy of the opponent. We study the evolution of cooperation in a population of 100 agents, each of which updates her strategy according to the myopic best response update rule.

The evolution is simulated with different types of agents, each of whom has a different betrayal factor. We find that different equilibria can be attained in the population by changing the number of agents having a certain betrayal factor. The simulation results support the claim that decreasing the feeling of being betrayed in a portion of agents, does not necessarily increase the level of cooperation in the population. In other words, there are some states, at which the population becomes robust to agents with a lower level of feeling betrayed. Hence, replacing some of the high-betrayal-level agents in the population with some low-betrayal-level agents, does not change the total number of cooperators in the population. However, this resistance of the population against agents with a low level of the feeling of being betrayed is only up to some point. That is where the number of the agents in the population with a lower level of the betrayal factor reaches the number of cooperators at the symmetric Nash equilibrium of a 2 × 2 symmetric game with the same payoff matrix assigned to the low-betrayal-level agents. After that point, the average cooperation-level in the population is raised by the replacement of some high-level-betrayal agents with some low-betrayal-level ones. However, by further increasing the number of these low-betrayal-level type of agents in the population, the selfishness of these even highly cooperative agents will force them to start to defect. They defect in order to maintain the total level of cooperation in the population at their desired level. In the future, we are interested in investigating the evolution of cooperation under the same conditions but when the imitative update rule is used by the agents.

We also propose two other models where the betrayal factor of an agent fluctuates as a function of the number of times she encounters a cooperator and the number of times she encounters a defector. In the first model, the agents update their strategies as before. In this case the population exhibits a more unstable behavior comparing to the previous cases. However, for the same initial strategies, different runs result in almost the same final state of the ratio of cooperators. In the second model, the agents update their strategies based on the number of encounters. In this case, an unstable behavior is observed. Starting from the same ratio of cooperators in the population, the ratio of cooperators can converge to different final states. We have however discussed how to lead the population to the state of all cooperators or all defectors by tunning the parameters in the function just mentioned.
